# Acclimation

**Published:** 1837

**Authors:** 


### Art. VII.—Acclimation—Temperance in the South—Medical College of Louisiana.*

When man changes his climate, he must change some of his habits. Professor Barton, in his Lecture on Acclimation, has undertaken to inquire what changes should be made by the emigrant from the North, when he comes to reside in Louisiana. To this inquiry, he has brought a good stock of medical erudition, and the experience of many years spent in the interior of Louisiana and in New Orleans. Without attempting an analysis of his lecture, we shall present his conclusions, as they seem to us to be just, and deserving of extensive dissemination.

"I have no hesitation in expressing my belief that the error of the counsel given to those going South, to employ stimulants to counteract the supposed debilitating influence of a warm climate, (which has no mean authority for its advocacy,) has arisen from confounding the effect of the long continuance of heat, with that of first impressions—the one bearing, often calling for stimulants—the other, (proceeding directly from the stimulus of heat,) adding to and heightening all the injurious effects of a hot climate. This fatal error has carried millions to the grave, and will continue to do so, until men reason rightly from effects to causes. Experience upon this subject lends its important aid, and confirms the value of the theory; a few references to illustrative authority will not be out of place."

The distinction recognized in this paragraph, is undoubtedly well founded. His views, in detail, will be seen in the follow-pages:

"There are certain Hygienic rules to be followed during the process of acclimation, which time permits me only very briefly to advert to—in the careful observance of which, individuals are enabled to resist morbific climatural influences they are unavoidably exposed to. They prescribe certain regulations with regard to DIET, DRINKS, CLOTHING, HABITS OF LIFE, &c.

"This is not the place to descend to much detail into these circumstances. They will come in more appropriately at an early period of our course. I shall then merely fill up, in a general manner, the outline I have sketched.

"The kind of *diet* required during the *process of acclimation* is clearly inferrible from the position laid down with regard to calorification, and has been, in general, mentioned. It is to be of the lightest

---

* Introductory Lecture on Acclimation delivered at the opening of the third session of the Medical College of Louisiana, by E. H. Barton, M. D., Professor, &c. New Orleans, 1837.

A Discourse on temperance and the applicability of stimulants in a warm climate, delivered before the New Orleans Temperance Society, by E. H. Barton, M. D. 1837.

kind, of plain unirritating vegetables, of little animal food during the first summer, and that fresh, and avoiding much indulgence in salt food. The advice of using salt food in hot weather, given by a distinguished professor, now no more, has cost thousands of lives, some to my own personal knowledge. The second summer a little more freedom may be indulged. After acclimation, a more generous living is advisable. I have little doubt that good living is conducive to health, as giving that degree of tone to the system that enables it to resist the influence of morbific causes.

"The same general principles are applicable to DRINKS, absolutely forbidding those of a stimulating nature, as increasing the range of those organic actions, and particularly those of the gastric system, already over-stimulated by the climate. Bland and acidulated drinks, (where they agree,) and the Seltzer water, are highly refreshing and proper. The consequences of indulging in stimulating drinks in a warm climate have been recently shown on another occasion.* The indulgence, particularly the intemperate use of ardent spirits, retards acclimation, multiplies the changes of febrile excitement, and lessens the prospect of passing the acclimating fever with safety. It has been shown in the address just referred to, that they act in a direct line with, and aggravate all the injurious influences of a hot climate and season. This influence was exhibited in a remarkable manner from the records of the Charity Hospital, (the only data of the kind in this country.) From these, it appears that the mortality in that house, in 1835, amounted to 1226; of these, 940, or four-fifths! were produced by intemperance, and that only nine of this number had passed through their acclimation! a more pregnant and condemnatory fact of the use of stimulating drinks in a hot climate could not be cited. I know of no circumstances in which they are *necessary* for health, whether from exposure to wet, heat, or fatigue, and believe them to be injurious and only a counterfeiter of good in all.

"CLOTHING must be adapted to circumstances, bearing in mind the general principles, and that the surface is the great avenue to disease. A corpulent individual will require less than a spare man. If exposed much to the sun with a liability to perspire freely, increased susceptibility is produced. Hence that kind of clothing is requisite, which, though light, is a bad conductor of caloric, for the heat of exposure to the direct rays of the sun is greater than that of the human body. If the occupation does not exact such an exposure, or loss of fluids by perspiration, then light cool clothing is clearly called for.

"With regard to the HABITS OF LIFE, the errors have not been less serious than extensive: I cannot stop to enumerate them in detail; they may be inferred from the advice I am about to give. *Early rising*, to enjoy the delightful freshness of the morning breeze, is highly conducive to health. An early breakfast is the only 'fortifying' the stomach requires.

"*Exercise* is of great importance in this climate. It should be taken during the early morning and evening hours, avoiding the mid-day sun. The night air is only injurious to those who have been exposed much to the sun and excessive perspirations during the day; otherwise it is grateful and safe, particularly if in motion, carefully avoiding the extent of chilliness. Probably there is no habit more universal,

* Vide Address "on the applicability of stimulants in a hot climate," by the Author.

or that would strike a stranger more forcibly on visiting this country for the first time in summer, than the general habit of setting out and indulging in the evening air, (and particularly in this city,) and with perfect safety, under the restrictions just laid down, in defiance of all the predictions of the miasmatists.

"There is no habit more eminently promotive of health and long life, and as a preventive to disease, than the free use of the BATH. Believing that the main avenue to climatural disease to be the surface, that its appearance is the main index to the condition of the individual —the protection of that surface—the fortifying it against the inroads of morbific impressions, which are principally through the medium of the atmosphere, to which it is constantly exposed—the removal or modification of that sensibility and impressionability, upon which its susceptibility depends, surely cannot be over-rated. This is done by the purifying and cleansing process of the bath—opening the clogged and exciting the torpid capillaries, giving tone to the surface by its temperature and quality, and by frictions with the flesh brush. They should be daily administered."

In his TEMPERANCE DISCOURSE, the Professor displays a commendable zeal, and an intimate acquaintance with the subject; which he treats physically, morally and statistically, in a manner that does him credit, and cannot fail to be useful, in a city where, according to his estimates, the expense of drinking amounts, annually, to near seven and a half millions of dollars! This sum, however, embraces the consequences of drinking. But our limits do not permit us to travel through his details; and we shall dismiss the subject, by transcribing the happy peroration with which his discourse is concluded:

"With what a brilliant vision do you clothe the future with regard to this portion of our happy country—what makes it so? You complete the outline in having the country filled with an enlightened population—the forest subdued—the land cleared, drained and cultivated—canals and railroads giving every facility of intercourse: but, you will have the same burning sun, the same vicissitudes, the same moisture—and will not the same habits produce the same effects then as now? Will not ardent spirits have the same influence in increasing the liability to climatural disease, and augmenting every malign influence of position? Your vision, then, of the future, instead of being of joyous anticipation, with the growing influence of this vice upon us, should be one of the most sombre melancholy; for increase of population, under such circumstances, can only produce increase of mortality and crime. But what a splendid contrast opens to the eye of the philanthropist and the christian of this country, with a change of habits and all the happy consequences I have shown to flow from it. What a delightful relief, to see the springs of life, feeling and intelligence renewed on every hand: health, industry and prosperity glowing around us; and the altars of domestic peace and love rekindled in every family. America and her institutions are the last hope of the world. It is one of the great resources left her for paying off, to the old world, the large debt incurred for the benefits she has derived from her literature, her arts, and her sciences. But I am at fault; my language is of the future, while my data are of the past—the set-off has been registered—the claim allowed—the acquittance granted. The debt is extinguished, and a heavy balance has been entered to the credit of American philanthropy and benevolence."

Of the progress of the MEDICAL COLLEGE OF LOUISIANA, we are not well informed. By a circular attached to the Lecture on Acclimation, we learn that the present professors, are Drs. STONE, BARTON, HARRISON, JONES, MACKIE, and RIDDELL; the last of whom, a chemist and botanist of solid science, was lately an adjunct in the Medical Department of the Cincinnati College, and is advantageously known to the readers of this Journal. The experiment of establishing a medical school, as far south as the latitude of New Orleans, has not before been made, since the reign of the Ptolemies, at Alexandria; where the *first* dissections of the *human* body were made by Herophilus and Erasistratus. Our friends in New Orleans, rest their hopes of success upon the extraordinary opportunities for dissection which that city affords. May their fame equal that of the philosophers of the Nile. D.

